# Parents’ awareness in the Kingdom of Saudi Arabia regarding failure to thrive: A descriptive cross-sectional study

**DOI:** 10.15537/smj.2022.43.12.20220511

**Published:** 2022-12

**Authors:** Reem A. Alshammari, Osama S. Alnezari, Waleed A. Alhirabi, Salm J. Alaamer, Abdulmajeed S. Alsadun, Abdulmalik F. Alhmazani, Yousef F. Bakrshoom, Ali H. Alharbi

**Affiliations:** *From the Department of Pediatric (Alshammari), University of Hail-Medical College, and from the College of Medicine (Alnezari, Alhirabi, Alaamer, Alsadun, Alhmazani, Bakrshoom, Alharbi), University of Hail, Hail, Kingdom of Saudi Arabia.*

**Keywords:** awareness, parents, failure to thrive, growth faltering, growth failure

## Abstract

**Objectives:**

To assess the level of parental Awareness about growth failure across all of Saudi Arabia and look into the potential influence of covariates (social and demographic) as determinants of the level of knowledge of parents, both mother and father, aged between 18 and 60, and on the national level.

**Methods:**

A cross-sectional study involving a survey of 4,404 parents, aged between 18 and 60 years, in all administrative regions within Saudi Arabia was undertaken From March 2022 to May 2022.

**Results:**

The majority of participants had average awareness of FTT, and differences in region, as well as in educational and professional levels, had an impact on this awareness, with participants from the western region who hold bachelor’s degree and employees have a better level of awareness than others.

**Conclusion:**

To raise awareness, the whole public, especially parents, has to be continuously informed on failure to thrive in children and how to deal with it through educational programs and awareness campaigns.

**W**hen height and weight measures are below the fifth percentile on the growth chart or fall down 2 or more major growth percentiles, it is considered to be physically failing to thrive.^1,2^ A child who is not developing in accordance with expectations is referred to as failing to thrive (FTT) in the pediatric community.^3^ The failure to thrive is linked to abnormal growth and development. The condition generally results from inadequate nutrition, though on occasions there may be alternate or additional contributory factors.^4^ For instance, malabsorption disorders like cystic fibrosis celiac disease or severe allergies are also significant causes of FTT. Additionally, people who have a genetic condition or congenital cardiac disease may need more calories than they think.^5,6^ Any such condition where a child needs more calories than assumed can in turn lead to FTT, too. In extreme cases, moreover, parental/guardian neglect or abuse can also cause FTT, with food either not provided or purposely withheld from an infant.^7-11^ The most common symptoms of FTT are poor weight gain, irritability, along with easy and excessive fatigue and sleepiness. In turn, generalized development delay can be one of the most significant consequences of FTT.^12^ Diagnosis in most cases involves regular measurements, observation and follow-ups for the concerned child. The factors which are needed to be considered include the child’s age, general health, past medical history, as well as the severity of their symptoms.^4,13^ A team of healthcare professionals, such as social workers, dietitians, physical therapists, geneticists, and other experts, are generally involved in treatment. When children do not get better and grow back to normal after therapy interventions, cooperation between many providers is crucial.^4^ As a result of the many effects and complications resulting from growth failure (FTT), including delayed growth and maturation, revealing the awareness of parents in Saudi Arabia on growth failure, its factors, causes and effects, is critical to protecting the health of mothers, children and infants. In addition, more studies are needed in Saudi Arabia that gauge parents’ awareness of failure to thrive carried out in Saudi Arabia that try to gauge how aware parents are of growth failure. As a result, we made the decision to assess the level of parental awareness on growth failure across all of Saudi Arabia and look into the potential influence of covariates (social and demographic) as determinants of the level of awareness of parents, both mother and father, aged between 18 and 60, and on the national level.

## Methods

The sample size was estimated using the Raosoft Sample Size Calculator, taking into account the expected response of 50%, the margin of error of 5%, and the confidence interval of 95%. As the minimum needed sample size is 385, and for a representative sample of survey respondents with minimal bias, we multiplied the sample size and added a 35% increment. Following the application of exclusion criteria, a representative sample (n=4,404) of parents participated in the survey from all regions of Saudi Arabia.

The inclusion criteria were parents, whether mother or father, aged between 18 and 60 years, in all administrative regions within the Kingdom. While the general population, and under the age of 18 and over 60 years of age is excluded. The study was carried out from March to May 2022.

A structured questionnaire was created by the researchers under the supervision of a pediatric teaching assistant and consultant pediatrician after they carried out a literature study and sought advice from other professionals in the field. The main survey included 6 questions (age, gender, nationality, region, level of education, and occupation) inquiring regarding the sociodemographic characteristics of the participating parents and 18 questions measuring the participating parents’ levels of FTT awareness. It contained (a general definition of growth failure, its causes, symptoms, factors and management, in addition to child nutrition).

### Statistical analysis

Following data extraction, it was then revised, coded and fed into statistical software for data analysis. Namely, SPSS version 22 (IBMCorp, Armonk, NY, USA). Descriptive analysis based on frequency and percent distribution was undertaken for all variables, including demographic data, and awareness items. In respect awareness items, 10 questions were surveyed, a correct answer scored 3 points. A total summation of the discrete scores of the different items was then calculated. Correct answers received a value of 3, incorrect 0. The average awareness was then calculated for each participant in the sample. Participants whose mean score was below 1.5 were considered to have poor awareness. Those who scored 1.5-2.5 were considered to have intermediate awareness, while good awareness was achieved if a participant scored 2.5 or above. Finally, all statistical analysis was done using 2 tailed tests. A Chi-squared test was used to calculate the *p*-value. A *p*-value of under 0.05 was considered statistically significant. The study results were presented via tables, graphs, and pie charts.

The questionnaire was pre-tested on 10 persons, randomly selected from different age groups, prior to the onset of the actual data collection process. The knowledge scale’s reliability test, which was based on the scoring result, produced an acceptable result with a Cronbach’s alpha of 0.720. Any problems that were identified in the pre-test were dealt with by amending the questions such that they became more concise and intelligible. The questionnaire was presented once to each respondent.

Ethical approval was obtained from the Medical Research Ethics Committee at Hail University (No: H-2022-155). Informed consent was obtained from all respondents at the start of the questionnaire.

## Results

A total of 4,404 participants from different regions of Saudi Arabia responded to the survey. Responses were collected from March 2022 to May 2022. All participants completed the survey, giving a response rate of 100%. The demographic information collected included age, gender, region, nationality, educational level, and occupation.

Table 1 displays the distribution of social and demographic data for the participants. As it appears that the distribution of participants at various ages is equal, as was intended to obtain responses from various ages in an equal manner. The majority of the participants were Saudi women from the western region who had attended university and were currently working, along with Table 2 showing the distribution of their level of awareness regarding failure to thrive. As expected, the majority of participants had an intermediate level of awareness, and social and demographic co-factors had a part in the level of awareness that was noticed. The χ^2^ analysis for awareness level and the socio-demographic variables showed significant (*p*<0.05) associations, except in respect age and gender. Considering the regional differences in awareness levels, a statistically significant difference between the participants’ educational status and their awareness level was discovered (*p*=0.000). In terms of the participants’ education level and awareness levels, parents with university degrees tend to have higher awareness levels. On the other hand, parents who had not received education were more likely to have low awareness levels. The participants’ education level and level of awareness were found to differ statistically (*p*=0.048). There was a statistically significant difference between the nationality of participants and their awareness level with regard to these 2 variables (*p*=0.008). As well there was a statistically significant difference between the functional status of the participants and their awareness level when it came to the occupation status of the participants and their levels of awareness (*p*=0.048).

**Table 1:** - General characteristics of the studied sample (N=4404).

Factors	n	%
***Age in year***		
15-30	1474	33.5
31-40	1389	31.5
41-50	1110	25.2
>50	431	9.8
***Gender***		
Female	3090	70.2
Male	1314	29.8
***Nationality***		
Non-Saudi	244	5.5
Saudi	4160	94.5
***Region***		
Central region	828	18.8
Eastern region	880	20.0
Southern region	988	22.4
Northern region	647	14.7
Western region	1061	24.1
***Education***		
primary school	125	2.8
Middle school	181	4.1
High school	728	16.5
University	2882	65.4
Postgraduate	394	8.9
Illiterate	94	2.1
***Occupation***		
Employed	2494	56.6
Unemployed	1910	43.4

**Table 2:** - Selected characteristics of the sample, and their association with the awareness level regarding failure to thrive.

	Awareness level of the participating parents			
Factors	Good awareness	Intermediate awareness	Poor awareness	*P*-value
***Age in year***				
15-30	30 (0.6)	975 (22.1)	469 (10.6)	0.583
31-40	30 (0.6)	926 (21.0)	433 (9.8)	
41-50	22 (0.4)	730 (16.5)	358 (8.1)	
>50	15 (0.3)	273 (6.1)	143 (3.2)	
***Gender***				
Female	65 (1.4)	2015 (45)	1010 (22.9)	0.171
Male	32 (0.7)	889 (20)	393 (8.9)	
***Region***				
Central region	63 (1.4)	604 (13.7)	161 (3.6)	0.000*
Eastern region	0	424 (9.6)	456 (10.3)	
Southern region	16 (0.3)	551 (12.5)	421 (9.5)	
Northern region	18 (0.4)	561 (12.7)	68 (1.5)	
Western region	0	764 (17.3)	297 (6.7)	
***Education***				
University	71 (1.6)	1892 (42.9)	919 (20.8)	0.000*
High school	12 (0.2)	472 (10.7)	244 (5.5)	
Postgraduate	9 (0.2)	265 (6)	120 (2.7)	
Middle school	4 (0.1)	117 (2.6)	60 (1.3)	
Primary school	0	74 (1.6)	51 (1.1)	
Illiterate	1 (0.02)	84 (1.9)	9 (0.02)	
***Nationality***				
Saudi	85 (1.9)	2685 (60.0)	1327 (30.1)	0.008*
Non-Saudi	11 (.2)	170 (3.0)	63 (1.4)	
***Occupation***				
Employed	55 (1.2)	1682 (38.0)	757 (17.1)	0.048*
Unemployed	42 (0.9)	1222 (27.0)	646 (14.6)	

*Significant

**Figure 1:**
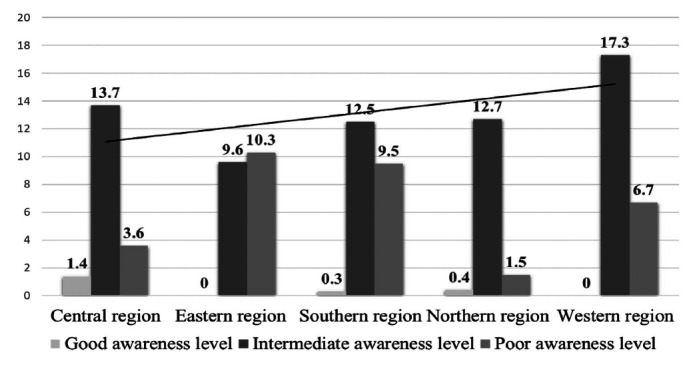
- The regional distribution of the awareness level regarding failure to thrive.

In respect the regional differences in awareness levels, 1.4% of participants from the Central region had a good awareness level. The Southern region has an awareness level of 0.4% and 0.3% in the Northern region. The samples from the Eastern and Western regions contained no participants with good awareness levels Figure 1.

**Figure 2:**
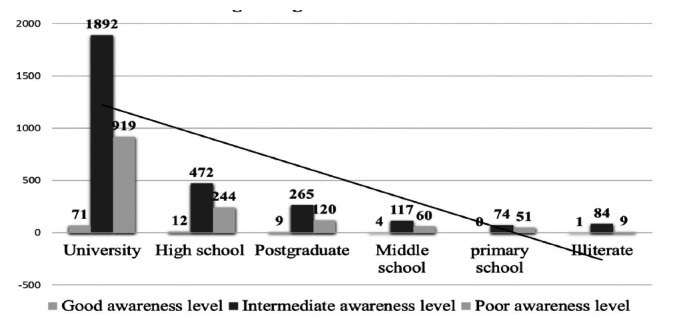
- The educational distribution of the awareness level regarding failure to thrive.

Regarding to the participants’ educational level and their awareness level, good awareness levels were mostly found in parents who have university education level. On the other hand, poor awareness levels were most common among non-university educated parents. Figure 2.

**Figure 3:**
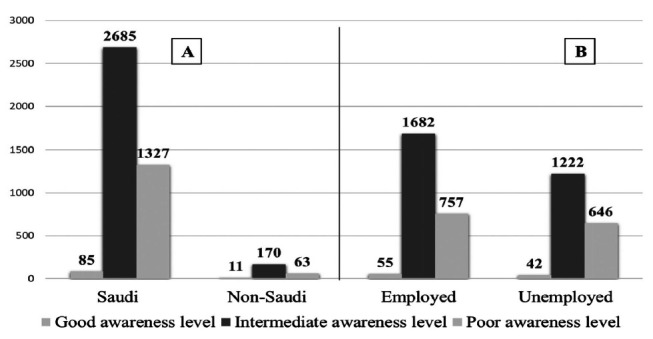
- The nationality and occupation status distribution of the awareness level regarding failure to thrive. **A**) Nationality distribution of the level of awareness of failure to thrive, **B**) Occupational distribution of the level of awareness of failure to thrive.

Regarding to the participant nationality and their awareness level, 1.2% of Saudi national parents, compared with 0.2% non-Saudi national parents, showed a good awareness level. Furthermore, 60% of Saudis compared with 3% of non-Saudis had an intermediate awareness level, while 30.1% of Saudis and 1.4% of non-Saudis had a poor awareness level (Figure 3A). While the occupation status of the participants and their awareness levels it was described in Figure 3B. The good awareness levels were identified among 1.2% of employed parents compared with 0.9% of unemployed parents. Intermediate awareness levels were reported in 38% of employed parents compared with 27% of unemployed parents. Last, poor awareness levels were found among 17.1% of employed parents and 14.6% of unemployed parents.

Table 3 shows the questions by which participants’ awareness of growth failure and the factors which cause it was measured.

**Table 3:** - Awareness-measuring questions about failure to thrive and its factors.

Factors	The correct answers	n	%
How do you know that your child is gaining the ideal weight for his age?	Go to medical visits and examine it and take growth indicators periodically	1499	34.0
Have you seen sources to explain the nutritional programs appropriate for your children	Yes	2948	66.9
Do you think that family history has anything to do with poor child’s weight?	No	694	15.8
What is the common cause of poor normal weight gain?	Malnutrition	2324	52.8
What are the factors that may lead to poor weight of the child?	Premature birth	1834	19.7
	Double weight at birth	1718	18.6
	Chronic diseases	2692	29.0
	Teen mother	916	9.9
	Educational level for child care	875	9.4
In Your opinion What is the right time to break the baby about milk feeding?	At the age of 2 years	2455	55.7
What is the most appropriate time to make food?	At the age of 4-6 months	2564	58.2
How much do you think your child needs to feed (rice, vegetables, fruit, white meat) before a year?	More than 50%	1461	33.2
How much do you think your child needs to feed (juice or milk domestic record) before a year?	More than 50%	1182	26.8
How can the number of meals and their quantity during the day can vary between babies and bulldozers?	The baby is given a few foods and a quantity, fish, textures, and gradual diversity with age	2184	49.6
Is it permissible to have the oldest age of the year and his meal with family?	Yes	2730	62.0
When a child loses or loses weight, what is appropriate to do, and in your opinion, a successful outcome for treating the condition?	Go to children’s clinic for medical consultation	362	8.2
The neglect of the child or child abuse can lead to weight decrease?	Yes	3753	85.2
Thyroid activity can lead to insufficient weight gain?	Yes	2722	61.8
What is the first signs of growth failure?	Weight loss	499	11.3
Failure to treat growth failure can affect mental abilities?	Yes	3058	69.4
Delay in giving vaccinations to the child can leads to failure to thrive?	No	1043	36.3
In the event of severe growth failure, is it possible for short stature and small head circumference to occur with weight loss?	Yes	2829	64.2

Values are presented as number and percentage (%).

## Discussion

The Saudi population’s knowledge of FTT and its causes was examined in this study. The objective was to comprehend the current level of community awareness and how to raise it further. This was important to do because parental thoughts and behaviors towards their children’s health and development are greatly improved by awareness. Since normal growth is an indication of good health in children.^14^ A good degree of parental awareness is helpful in monitoring growth, early discovery of the causes of poor development, and early intervention, all of which increase the likelihood that the kid will have good health. Second, a prior study by Hoddinott et al^15^ demonstrated that growth failure has a major impact on crucial factors, including family formation, reproduction, men’s pay rates, the prevention of poverty, education, and cognitive success. This emphasizes the significance of fostering linear growth from conception to age 2 and enhancing childhood nutrition because these actions benefit both individuals and their families for the rest of their lives.^15^ The importance of this study is that asses the level of awareness of parents on a national scale to test parental awareness on a national scale.

When investigating the parents’ awareness levels, through a set of questions, it was found that most of the participants had moderate levels of awareness on failure to thrive in children, which indicates that raising public awareness of failure to thrive is necessary in Saudi Arabia, where many regions call for particular attention. In addition, the studies’ findings indicated that there are variations in awareness levels between educational levels, between Saudis and non-Saudis, and between employees and non-employees, which calls for additional and deeper research for deeper study on the subject, and more an effort to raise awareness on all fronts. Especially since there are no previous studies that presented changes in the levels of community awareness in the past, to monitor levels of awareness in the future and work on them.

When determining the participants’ degree of awareness, a series of questions was put to them that brought to light several crucial details that could significantly alter the health of the child. The reason of FTT is discovered by testing, imaging, and endoscopy in less than 1.4% of instances.^16^ Therefore, these assessments should only be performed on children who exhibit obvious signs of an organic condition and those who do not grow after receiving behavioral or dietary therapies.^17^ The results of the study indicated that 52.8% of parents think that malnutrition is the most common cause of FTT. Meanwhile, regarding the type of food provided to the child during the first year, our study found that 43.9% of infants receive less than 50% of juice and milk artificial sweeteners perhaps. It’s crucial to comprehend this since excessive consumption of nutrient-poor liquids, including so-called “fruit” juice, which is primarily flavored sugar water, causes satiety before nutrient-rich foods can be consumed.^18^ The study results shows that overall FTT awareness levels need to be improved in Saudi Arabia, with several regions showing a particular need for attention. Additionally, the majority of parents in this survey stated that they base their estimation of their child’s weight on how they perceive their child’s body shape and rely on seeing their child’s body shape to do so. New, culturally appropriate models must be developed in order to relate to the child’s weight status in the context of the Saudi community. The results also highlight there is a need to correct the misapprehension that family history is related to the child’s potential to gain weight. There is also a need to start introducing food at age of 4-6 months, rather than the common misconception of 9 months that most participants highlighted. Moreover, more FTT awareness programs are also needed. These factors are however merely the tip of the iceberg in respect improving FTT awareness levels in Saudi Arabia. The major emphasis must be on combating the misinformation that many parents’ encounter, by providing them with better access to verified healthcare sources and limiting the spread of less credible materials. The study results also indicate that most parents are unaware of the ideal weight for their children. Most (52.7%) determined their child’s weight from observation of their body shape and growth, rather than arranging medical visits and assessing growth indicators periodically. Most (51.8%) of those surveyed from the Eastern region displayed a poorer FTT awareness level than was the case with participants from the other regions. The region with the highest awareness levels was the Central region, where 7.6% of those surveyed had good awareness. The present study therefore accords with other research in regard the geographic differences in awareness levels which exist between Saudi regions. The study also accords with Al-Qahtani et al^18^ who claimed, that the majority of parents base their assessment of their children’s weight gain on how their children’s bodies seem. Furthermore, the study found that a correlation exists between educational level and awareness level. That is, the higher the participant’s educational level, the better their awareness level. The study also found that, when faced with poor weight gain in their child, 8.3% of parents first respond by purchasing multiple vitamins from the pharmacy, though without a specific idea of what deficiencies need tackling seemed to be Saudi. Furthermore, 85.2% of the parents surveyed agree that child abuse or neglect can cause FTT, due to the possibility of clinical bonding difficulties in infants with FTT. Therefore, pediatricians should think on seeking out further advice from mental health specialists who can help them assess the bond between an infant and its caregiver.^19^

### Study limitations

A cross-sectional method precluded the drawing of any inference on the causality between variables. Moreover, awareness levels were also categorized in 3 classes. This, however, may obscure differences between the 3 levels, so any generalizing of the study results should be carried out with caution. Notwithstanding these limitations, this study addresses a crucial health issue for the Saudi society. Its strengths lie in the random stratifying sampling methodology and the large general pediatric population that was obtained.

In conclusion, according to the investigation into the parents’ awareness levels, the majority of the parents who had a bachelor’s degree and were had occupations. This suggests that cognition has a strong foundation and that education and occupation play a big part in it. It is advised to undertake in-depth studies on the levels of awareness in each region and to conduct future research as there were disparities in the levels of awareness between the regions of the Kingdom, but the explanation for these differences is unclear. Besides that, the study’s findings indicate that attention should be provided to the ongoing activation of educational programs and awareness campaigns for various age groups via the Internet and the media alongside one another in health centers, public spaces, workplaces, schools, and universities in order to raise the level of awareness of FTT in the Saudi community.
